# Transcutaneous electrical nerve stimulation to reduce pain in post-op thoracotomy patients: A physical therapists’ perspective

**DOI:** 10.4103/0019-5049.71024

**Published:** 2010

**Authors:** Abraham Samuel Babu, Lenny T Vasanthan, Arun G Maiya

**Affiliations:** Department of Physiotherapy, Manipal College of Allied Health Sciences, Manipal University, Manipal, Karnataka, India

Sir,

Electrotherapy is a part of regular physical therapy practice which forms a main portion of both undergraduate and postgraduate education. It plays a role in providing pain relief for patients with both acute and chronic pain. In this era where a lot of emphasis is placed on appropriate drug management, it is commendable that the authors in their recent study[1] decided to use an adjunctive form of therapy like transcutaneous electrical nerve stimulation (TENS). The inclusion of a placebo group helped in the identification of the true effect of TENS so that the “Hawthorne effect” was neutralised. However, there are a few salient points that we would like to highlight from the perspective of the physical therapist.

TENS is a modality whose effects depend on the intensity used and also on the sensory and motor effects. With regard to this, even a low intensity of TENS could produce an effect on the nerves in the control group. Literature describes an intensity which is comfortable to the patient at a frequency between 80 and 100 Hz.[2] Clarity with regard to these parameters would help physical therapists manage pain among these patients after cessation of epidural analgesia.

Secondly, the authors mentioned that TENS was used to reduce the usage of adjunctive drug management. Solak *et al*. found that TENS did reduce the rescue medication needed 5 days postoperatively when compared with patients on controlled morphine.[3] If these findings can be proved in larger trials, the use of TENS can definitely be made a routine practice. Another benefit which may be in favour of the patient is probably the reduction in cost of the analgesic drugs. However, this is purely speculative and will need further research.

Thirdly, with regard to outcomes, we would like to highlight those that we think would be reliable for these patients. They are pulmonary function testing (PFT) as an indicator for airway clearance and patient comfort during activities of daily living (ADL). PFTs have been used in the literature as an outcome measure and have shown improvements with TENS.[4,5] It would also be worth obtaining information from other health care members regarding the degree of cooperation of the patient to airway clearance techniques, mobilisation and ADL.

Lastly, we believe it would be of great use to the patient if TENS was used in the later stages, especially after the discontinuation of epidural analgesics, as at this point pain forces the patients to be less cooperative with airway clearance techniques and mobilisation. If this occurs, the chances of developing pulmonary complications are high. However, the optimal timing for starting TENS among these patients needs to be determined.

To conclude, the use of TENS should be neither underestimated nor disregarded during the postoperative phase of patients undergoing thoracotomies. The positive results obtained in the study by Chandra *et al*.[1] and also in previous studies[4,5] show that TENS will be of benefit to the patient and also to the physical therapist to facilitate airway clearance and early mobilisation.
